# Substance Use Descriptive Norms and Behaviors among US College Students: Findings from the Healthy Minds Study

**DOI:** 10.3390/epidemiologia3010005

**Published:** 2022-01-27

**Authors:** Hans Oh, Megan Besecker, Jimi Huh, Sasha Zhou, Susan E. Luczak, Eric R. Pedersen

**Affiliations:** 1 Suzanne Dworak Peck School of Social Work, The University of Southern California, Los Angeles, CA 90007, USA; meganbesecker@gmail.com (M.B.); jimihuh@usc.edu (J.H.); eric.pedersen@med.usc.edu (E.R.P.); 2 Institute for Addiction Science, University of Southern California, Los Angeles, CA 90007, USA; 3 Public Health Department, Wayne State University, Detroit, MI 48202, USA; sashaz@wayne.edu; 4 Department of Psychology, University of Southern California, Los Angeles, CA 90007, USA; luczak@usc.edu

**Keywords:** alcohol, tobacco, cannabis, vaping, norms

## Abstract

Background: Social norms have been associated with alcohol use in college populations; however, more research is needed to confirm the associations between social norms and a range of substance use behaviors during the COVID-19 pandemic. Methods: We analyzed data from the Healthy Minds Study (September 2020–December 2020), a non-probability sample administered online to college students. We used multivariable logistic regression to test for associations between respondents’ perceptions of substance use behaviors in their respective colleges and their own substance use behaviors, adjusting for age, gender, race/ethnicity, and international student status. Results: We found that those who overestimated the prevalence of alcohol use, cigarette use, cannabis use, and vaping were significantly more likely to use these substances when compared with those who did not overestimate. These associations persisted even when using different prevalence estimates of substance use, though some associations lost statistical significance when applying the survey weights to account for non-response. Conclusion: College students overestimated the prevalence of substance use in their respective colleges, even during the early stages of the pandemic when social interactions were limited, and these beliefs were associated with substance use. Future studies may test the utility of campaigns to alter perceptions of social norms and interventions that use personalized normative feedback to reduce substance use during pandemics.

## 1. Introduction

Perceived norms are beliefs that individuals hold about the behaviors of their peers. One specific type of perceived norm is the descriptive norm, which is how one perceives the typical patterns of behavior in a given peer group, coupled with the expectation that people will behave according to these patterns. A substantial body of research has shown that perceived norms about alcohol use are associated with actual drinking behaviors among college students [1,2,3,4,5]. That is, college students tend to overestimate how many of their peers engage in heavy alcohol consumption [4], and these exaggerated views putatively act as a causal factor of heavy drinking. As a result, interventions have sought to change misperceptions of peer drinking norms [6,7,8]. This line of research has also extended to other substances, including tobacco use [9], and cannabis use [10,11], though the body of evidence is relatively smaller for these substances [12]. Still, correcting the misperceptions of peer norms during college may help individuals form more accurate perceptions of substance use, which may then help decrease future use.

The COVID-19 pandemic impacted the entire globe throughout the year 2020 and beyond in various domains of life, including substance use, though studies found mixed evidence on whether substance use increased or decreased during this time period [13,14]. Regardless, the pandemic created a unique opportunity to examine social norms and the extent to which they influence behaviors in the absence of social interactions. The COVID-19 pandemic was particularly disruptive for university student populations [15]. One study found that among a sample of college students (N = 507), most students reported decreased alcohol use and perceived that their peers also decreased alcohol use; the perceptions of peers’ changes in alcohol use behavior strongly related to changes in students’ own alcohol use [14]. Reductions in alcohol use may have been attributed to changing social environments (e.g., moving back home to live with parents; [16]. Research has also suggested that aspects of the pandemic (specifically stay-at-home orders and social isolation) may have resulted in an uptick in cannabis use and tobacco use for some individuals yet may have deterred use due to reduced social interactions and increased fears of sharing products [17,18]. Further, the social nature of vaping has been documented in literature [19,20,21], and this may have been disrupted during the pandemic.

While a sizable body of research has been conducted on social norms and alcohol use among college students, there is relatively less research on tobacco and cannabis use, and scarcely any research on vaping. Moreover, social norms theory posits that people form and reinforce their perceptions through observation, but there are few studies that have examined whether the association between perceptions and substance use behaviors is stable during times of crisis. To fill these gaps, we analyze a sample of college students, and in doing so, we examined whether perceived norms were related to substance use behaviors despite reduced social contact during the early stages of the COVID-19 pandemic.

## 2. Methods

### 2.1. Sample

We analyzed data from the Fall semester cohort of the 2020 Healthy Minds Study (HMS), a cross-sectional, web-based survey examining mental health and related factors in undergraduate and graduate student populations. The survey was administered at 36 colleges across the country between September 2020 and December 2020. At each college, a random sample of 8000 students was invited by e-mail to participate, except at smaller colleges (<8000 students) where all students were invited to participate. Five colleges elected to administer the substance use module, yielding a final analytic sample of N = 2115. The Healthy Minds Study was approved by Advarra, and by the institutional review boards at each participating college.

### 2.2. Measures

#### 2.2.1. Substance Use Behaviors 

Alcohol use was measured using the item: Over the past 2 weeks, did you drink any alcohol (yes/no)? Cigarette use was measured using the item: Over the past 30 days, about how many cigarettes did you smoke per day? Respondents could answer: 0 cigarettes, less than 1 cigarette, 1 to 5 cigarettes, about one-half pack, or 1 or more packs. Due to small cell counts, this frequency variable was dichotomized into a single variable to reflect any cigarette use over the past 30 days. Cannabis use was measured using the dichotomous item (yes/no): Over the past 30 days, have you used marijuana? Vaping was measured using the single dichotomous item (yes/no): Over the past 30 days, have you used an electronic cigarette or vape pen? Vaping was defined broadly to include nicotine, cannabis, and/or flavoring.

#### 2.2.2. Perceived Substance Use Norms 

Perceived substance use norms were measured using four items that asked: “In the past 30 days, about what percent of students at your school…” Respondents were asked to estimate the percentage of the student body who drank alcohol, smoked cigarettes, smoked (or otherwise used) marijuana, and vaped. To establish a cut-off to determine whether respondents overestimated substance use behaviors in their respective schools, we used the proportions of substance use drawn from the data collected at each college. We then created a dichotomous variable to reflect whether students over-estimated substance use in their schools based on this cut-off.

#### 2.2.3. Sociodemographic Covariates

Respondents self-reported sociodemographic characteristics, including age (continuous), gender (man, woman, transgender/nonbinary/other), race/ethnicity (White, Black, Latinx/Hispanic, Asian American/Pacific Islander, Multiracial, Other), and international student status (yes/no).

### 2.3. Analysis

Multivariable logistic regression analyses were used to test for associations between respondents’ perceptions of substance use behaviors in their respective colleges and the respondents’ own substance use behaviors, adjusting for age, gender, race/ethnicity, and international student status. Perceptions of substance use were treated continuously and dichotomously. To determine whether respondents overestimated the prevalence of substance use, we established cut-offs based on the prevalence reported at each school. Missing data for individual variables were handled using listwise deletion, which is appropriate for these survey data given the low frequency of missingness (<5%). Sample probability weights were used to adjust for non-response. Standard errors were clustered by college. We used sample probability weights to adjust for non-response using administrative available data on full student populations at each institution. Using multivariable logistic regression, response propensity was estimated based on gender, race/ethnicity, academic level, and grade point average. We then assigned response propensity weights to each student who completed the survey. Students who were less likely to have completed the survey were assigned a larger weight in the analysis. Sample weights gave equal aggregate weight to each school in the national estimates rather than assigning weights in proportion to school size so that overall national estimates were not dominated by schools in our sample with large enrollment. However, only five colleges administered the items on perceived descriptive norms. To account for the inherent assumptions made in the creation of survey weights, we provide all unweighted models as well. Standard errors were clustered by college. All results were presented as odds ratios with 95% confidence intervals. Analyses were conducted using Stata SE 15.

### 2.4. Sensitivity Analyses

We conducted two sensitivity analyses using cut-offs based on the prevalence established in prior survey data (which likely captures the prevalence of substance use before the pandemic) [22,23,24] and cut-offs based on the entire HMS dataset (which may capture the prevalence of substance use during the pandemic based on 34,115 students from across 36 colleges). Sensitivity analyses are presented in the Supplementary Materials.

## 3. Results

Among the sample who received the measures used in this study (N = 2115), most of the weighted sample was White (80.47%), the majority were men (56.15%), most were domestic students (96.89%), with an average age of approximately 23 years old. Tables S1 and S2 in the Supplementary Materials provide the descriptive statistics for the weighted and unweighted prevalence of substance use and prevalence of overestimation of substance use at each of the five colleges included in this study. Across all five colleges, 46.5% (45.5% unweighted) of students used alcohol over the past two weeks, 7.3% (5.6% unweighted) used cigarettes over the past month, 13.7% (11.7% unweighted) used cannabis over the past month, and 17.8% (13.7% unweighted) vaped over the past month. Using school-specific cut-offs, 59.8% (55.7% unweighted) overestimated the prevalence of alcohol use, 76.1% (75.5% unweighted) overestimated the prevalence of cigarette use, 77.1% (79.2% unweighted) overestimated the prevalence of cannabis, and 73.4% (78.6% unweighted) overestimated the prevalence of vaping. Additional prevalence estimates are provided in Table S1 based on the larger HMS dataset (which spans 36 colleges in the United States) and based on prior studies [22,23,24].

When coding perceived use continuously, a one-percentage increase in estimation of alcohol use at their own school was associated with 3% higher odds of using alcohol (weighted aOR:1.03, 95% CI:1.02, 1.03; unweighted aOR: 1.03, 95% CI: 1.03–1.04). Overestimating alcohol use among their own school was associated with 2.42 times greater odds of consuming any alcohol when compared with those who did not overestimate. A one-percentage increase in estimation of cigarette use at their own school was associated with 2% higher odds of using cigarettes (weighted aOR: 1.02; 95% CI: 1.00, 1.03, unweighted aOR: 1.02, 95% CI: 1.01, 1.03). Overestimating cigarette use was associated with 1.81 times greater odds of any cigarette use over the past month when compared with those who did not overestimate, though this did not reach a conventional level of statistical significance in weighted models. A one-percentage increase in estimation of cannabis use at their own school was associated with 3% higher odds of using cannabis (weighted aOR:1.03; 95% CI: 1.01, 1.05, unweighted aOR: 1.04, 95% CI: 1.03, 1.05). Overestimating cannabis use in one’s own school was associated with 3.89 times greater odds of any cannabis use over the past month when compared with those who did not overestimate. A one-percentage increase in estimation of vaping at their own school was associated with 2% higher odds of vaping (weighted aOR: 1.02; 95% CI: 0.98, 1.05; unweighted aOR: 1.03, 95% CI: 1.02, 1.04). Overestimating how many students vaped in one’s own school was associated with 1.32 times greater odds of any vaping over the past month when compared with those who did not overestimate. Results of multivariable logistic regression models are presented in Table 1. All multivariable logistic regression models were replicated using the prevalence of substance use found in prior studies, and using the prevalence found in the larger HMS (36 colleges, N = 34,115), which can be found in Table S3.

## 4. Discussion

### 4.1. Main Findings

We found that during the COVID-19 pandemic, a significant percentage of college students overestimated the percentage of students at their respective colleges who used substances and that those who overestimated were more likely to engage in substance use than those who did not overestimate. This was true for alcohol use, cigarette use, cannabis use, and vaping, using various cut-offs based on college-specific prevalence estimates, national prevalence estimates prior to the COVID-19 pandemic, and the prevalence found in the larger HMS data collected during the pandemic. Not all substances reached a conventional level of statistical significance when survey weights were applied. Overall, these findings comport with existing literature that shows perceived descriptive norms are a strong predictor of actual substance use. A novel aspect of this study is that we show that most students overestimated vaping prevalence and that this overestimation was associated with significantly greater odds of vaping, which to our knowledge has rarely been studied. While the design of the study was cross-sectional and could not describe the impact of COVID-19 on substance use norms, we did find that descriptive norms are evident during an exogenous shock like the pandemic, when a complete upheaval of day-to-day life resulted in reduced social contact with peers via lockdown restrictions, thereby limiting opportunities to observe their peers using substances. Yet despite fewer opportunities to use substances (i.e., absence of parties, closed alcohol outlets, residing with parents while under legal age), a significant portion of the sample used substances over the past month. Even though overall use may have been limited during the lockdown, a significant number of students still overestimated their peers’ use, and these individuals were more likely to use substances themselves. This suggests that the effect of perceived norms on actual behavior may be durable throughout global crises [14,16].

### 4.2. Limitations

Our study should be interpreted bearing in mind several limitations. First, the data were cross-sectional, and we were unable to ascertain whether perceived descriptive norms caused more substance use, or if substance use shaped perceived norms. More longitudinal research is needed to strengthen causal inferences. Second, the response rate for this survey was 14%, which is low but consistent with other large online surveys of this nature [25,26]. Still, selection bias is a concern. We applied survey weights to account for non-response; however, this may have made estimates less precise. Future research can use probability sampling with incentives to allow for higher response rates and greater generalizability. Third, we used a general descriptive norm where the referent was the college student population. However, norms can vary by culture and context. We did not have sufficient power to detect effect modification; thus, more research is needed to test the interaction of norms with race/ethnicity, gender and sexual identity, and acculturation level. Fourth, there are other types of norms, such as injunctive norms (beliefs about how one should behave) that may have influenced substance use behaviors but were not captured in our study. A more comprehensive assessment of the various norms and the cultural variation of norms is needed. Finally, drug policies (e.g., the legalization of cannabis) and public health responses to the pandemic may have varied widely across states and may have influenced the likelihood of using substances and descriptive norms of substance use.

## 5. Conclusions

Social norms may play a key role in early interventions during emerging and young adulthood. Thus, correcting normative misperceptions (e.g., through personalized normative feedback) has been a promising strategy to modestly reduce alcohol use [12], and potentially other substances such as cigarettes, cannabis, and vaping among college students. During the COVID-19 pandemic, a significant portion of college students overestimated the prevalence of substance use. Future research can explore the various types of norms and how these norms influence substance use behaviors within various socio-historical and socio-cultural milieus. Targeting norms among college students and young adults may be critical given the amount of influence norms have on substance use behaviors during this life stage, in which intervention can potentially alter the trajectory of substance use into the future.

## Figures and Tables

**Table 1:** Multivariable logistic regression models showing the association between overestimation of substance use at one’s school and substance use, Healthy Minds Study, September 2020–December 2020.

	Weighted		Unweighted	
	aOR (95% CI)	*p*-value	aOR (95% CI)	*p*-value
Perceptions of alcohol use in the college				
Overestimated	2.42 (2.10, 2.80)	<0.001	3.65 (3.00, 4.45)	<0.001
N	2109			
Perceptions of cigarette use in the college				
Overestimated	1.81 (0.47, 7.00)	0.293	2.63 (1.34, 5.17)	0.005
N	2093			
Perceptions of cannabis use in the college				
Overestimated	3.89 (0.76, 20.02)	0.083	4.09 (2.34, 7.14)	<0.001
N	2098			
Perceptions of vaping in the college				
Overestimated	1.32 (0.32, 5.42)	0.609	1.97 (1.30, 2.98)	0.001
N	2078			

Ad justed for age, sex, race/ethnicity. Reference groups– those who did not overestimate substance use. Cut-off based on each college.

## Data Availability

The Healthy Minds Study data can be found at: https://healthymindsnetwork.org/hms/, accessed on 29 November 2021.

## Supplementary Materials

The following are available online at https://www.mdpi.com/article/10.3390/epidemiologia3010005/s1, Table S1: Prevalence of substance use; Table S2: Descriptive statistics; Table S3: Multivariable logistic regression models showing the association between overestimation of substance use at one’s school and substance use, Healthy Minds Study, September 2020–December 2020.
