# Supplementary material for: Substance Use Descriptive Norms and Behaviors among US College Students: Findings from the Healthy Minds Study
Source: Epidemiologia (Basel). 2022 Jan 27;3(1):42–8. doi: 10.3390/epidemiologia3010005 (PMC9620896; doi:10.3390/epidemiologia3010005)
Supplement: Supplementary file 1 [file epidemiologia-03-00005-s001.zip › epidemiologia-1512252-supplementary.pdf]

SUPPLEMENTARY MATERIALS

Table S1. Prevalence of substance use

	Colleges included in the study (based on 5 colleges)		All colleges in the HMS (based on 36 colleges)		National estimates from prior studies
	Weighted %	Unweighted %	Weighted %	Unweighted %	Unweighted%
Substance use					
Any alcohol use (past two weeks)	46.5%	45.5%	48.6%	49.3%	52.5%
Any cigarette use (past month)	7.3%	5.6%	7.2%	5.8%	19.1%
Any cannabis use (past month)	13.7%	11.7%	16.9%	16.4%	20.2%
Any vaping (past month)	17.8%	13.7%	14.5%	13.1%	22%
Perceived use					
Overestimation alcohol use	59.8%	55.7%	60.3%	49.7%	50.1%
Overestimation cigarette use	76.1%	75.5%	72.6%	67.2%	50.5%
Overestimation cannabis use	77.1%	79.2%	65.8%	55.6%	60.2%
Overestimation vaping	73.4%	78.6%	72.3%	68.4%	65.8%

Table S2. Descriptive statistics

	Weighted			Unweighted		
	N (yes)	%	Subpop size	N (Yes)	%	Total N
**College 1**						
Substance use						
Any alcohol use	412.5	49.41%	834.8	1,171	53.64%	2183
Any cigarette use	46.85	5.76%	813.8	125	5.87%	2131
Any cannabis use	138.9	13.86%	1002	356	13.90%	2562
Any vaping	118.9	14.66%	810.6	308	14.48%	2127
Perceived use						
Overestimation alcohol use	103.1	71.19%	144.8	292	74.68%	391
Overestimation cigarette use	119.2	82.29%	144.8	329	84.14%	391
Overestimation cannabis use	122.8	84.72%	144.9	339	86.70%	391
Overestimation vaping	119	82.35%	144.5	327	83.85%	390
**College 2**						
Substance use						
Any alcohol use	277.1	30.68%	902.9	102	33.33%	306
Any cigarette use	79.19	9.36%	846.2	27	9.41%	287

Any cannabis use	103.7	10.31%	1005.9	36	10.62%	339
Any vaping	165.3	19.54%	846.2	52	18.12%	287
Perceived use						
Overestimation alcohol use	407.3	61.56%	661.7	140	63.35%	221
Overestimation cigarette use	500.5	75.65%	661.7	171	77.38%	221
Overestimation cannabis use	476.7	72.05%	661.7	165	74.66%	221
Overestimation vaping	438.1	66.21%	661.7	155	70.14%	221
**College 3**						
Substance use						
Any alcohol use	539.1	59.50%	906	739	60.67%	1218
Any cigarette use	72.14	8.13%	887.7	86	7.20%	1195
Any cannabis use	174.9	17.43%	1005.1	224	16.65%	1345
Any vaping	174.5	19.69%	885.9	229	19.20%	1193
Perceived use						
Overestimation alcohol use	186.8	65.37%	285.7	256	66.67%	384
Overestimation cigarette use	253.5	88.73%	285.7	349	90.89%	384
Overestimation cannabis use	237.4	83.23%	285.2	330	86.16%	383
Overestimation vaping	226.9	79.56%	285.2	316	82.51%	383
**College 4**						
Substance use						
Any alcohol use	263.7	27.78%	949.3	220	17.36%	1267
Any cigarette use	38.52	4.27%	902.4	29	2.38%	1220
Any cannabis use	9.548	0.95%	1009.1	15	1.14%	1321
Any vaping	27.3	3.04%	898.8	47	3.88%	1211
Perceived use						
Overestimation alcohol use	303.1	43.89%	690.5	414	40.95%	1011
Overestimation cigarette use	432.7	62.81%	688.9	655	65.04%	1007
Overestimation cannabis use	470.2	68.22%	689.3	744	73.81%	1008
Overestimation vaping	486.1	70.52%	689.3	775	76.88%	1008
**College 5**						
Substance use						
Any alcohol use	572	67.88%	842.7	88	67.18%	131
Any cigarette use	74.8	9.42%	794.5	11	8.94%	123
Any cannabis use	258.9	25.89%	1000	39	25.16%	155
Any vaping	267.8	33.99%	787.9	41	33.61%	122

Perceived use						
Overestimation alcohol use	482.7	69.25%	697.1	75	69.44%	108
Overestimation cigarette use	580.4	83.26%	697.1	90	83.33%	108
Overestimation cannabis use	603.3	86.54%	697.1	93	86.11%	108
Overestimation vaping	547.5	78.54%	697.1	86	79.63%	108

Table S3: Multivariable logistic regression models showing the association between overestimation of substance use at one's school and substance use, Healthy Minds Study, September 2020 – December 2020

	Cut-off based on each college				Sensitivity Analysis 1: Cut-off based on national estimates				Sensitivity Analysis 2: Cut-off based on the entire HMS data			
	Weighted		Unweighted		Weighted		Unweighted		Weighted		Unweighted	
	aOR [95% CI]	P-value	aOR [95% CI]	P-value	aOR [95% CI]	P-value	aOR [95% CI]	P-value	aOR [95% CI]	P-value	aOR [95% CI]	P-value
Perceptions of alcohol use in the college												
Overestimated	**2.42 [2.10, 2.80]**	**<0.001**	**3.65 [3.00, 4.45]**	**<0.001**	**4.18 [3.08, 5.68]**	**<0.001**	**5.56 [4.58, 6.76]**	**<0.001**	**3.93 [3.23, 4.77]**	**<0.001**	**6.07 [4.96, 7.42]**	**<0.001**
N	2109											
Perceptions of cigarette use in the college												
Overestimated	1.81 [0.47, 7.00]	0.293	**2.63 [1.34, 5.17]**	**0.005**	**2.24 [1.45, 3.46]**	**0.007**	**2.40 [1.52, 3.80]**	**<0.001**	2.00 [0.53, 7.61]	0.223	**3.10 [1.66, 5.78]**	**<0.001**
N	2093											
Perceptions of cannabis use in the college												
Overestimated	3.89 [0.76, 20.02]	0.083	**4.09 [2.34, 7.14]**	**<0.001**	**16.5 [4.60, 59.24]**	**0.004**	**14.24 [8.69, 23.34]**	**<0.001**	**12.63 [3.21, 49.70]**	**0.007**	**11.11 [6.70, 18.43]**	**<0.001**
N	2098											
Perceptions of vaping in the college												
Overestimated	1.32 [0.32, 5.42]	0.609	**1.97 [1.30, 2.98]**	**0.001**	3.25 [0.92, 11.47]	0.060	**5.06 [3.40, 7.53]**	**<0.001**	2.96 [0.70, 12.52]	0.105	**5.45 [3.41, 8.74]**	**<0.001**
N	2078											
Adjusted for age, sex, race/ethnicity<br>Reference groups – those who did not overestimate substance use												
